# 

**Published:** 1883-02

**Authors:** 


CONTENTS:

PAGE.

ORIGINAL COMMUNICATIONS:

Cancer of the Neck of the Womb Treated by Scraping — Douglas's Cul-de-sac Opened—Recovery without a Bad Symptom—Abdominal Enlargement Probably due to Hysteria. By William Goodell, M. D. . . . . . . . . . 289

Some Points of the Histology of Malignant Tumors. By M. D. Mann, M. D. . 300

CLINICAL REPORTS:

A Clinical Lecture Delivered at the Hospital of the Sisters of Charity, Jan. 13th, 1883. By W. S. Tremaine, M. D., U. S. A., . 307

Erysipelas Occuring with Parturition. Reported by Bernard Bartow, M. D., . 318

SELECTIONS:

The Pyrogallic Acid Treatment of Phagedenæ, . . . . . . . . . 321

Hydrastin in Laryngeal Phthisis, . . . 322

CORRESPONDENCE: . . . . . 322

PAGE.

SOCIETY REPORTS:

Buffalo Medical and Surgical Association, . 323

Erie County Medical Society, . . . . 327

EDITORIAL:

Directory for Nurses, . . . . . . . 331

The Erie County Medical Society and the New Code, . . . . . . . 332

Editorial Notes, . . . . . . . 333

REVIEWS:

The Diseases of Women; their Pathology, Diagnosis and Treatment, including the Diseases of Pregnancy, . . 334

Electricity in Medicine and Surgery, . . 334

On Slight Ailments; their Nature and Treatment, . . . . . . . . 335

The Diseases of the Rectum, . . . . 335

A Practical Labratory Course in Medical Chemistry, . . . . . . . 336

The Hospital Treatment of Diseases of Heart and Lungs, . . . . . . 336

